# Community-level prevalence of epilepsy and of neurocysticercosis among people with epilepsy in the Balaka district of Malawi: A cross-sectional study

**DOI:** 10.1371/journal.pntd.0010675

**Published:** 2022-09-15

**Authors:** Luise Keller, Dominik Stelzle, Veronika Schmidt, Hélène Carabin, Ann-Kristin Reinhold, Claudius Keller, Tamara M. Welte, Vivien Richter, Action Amos, Lindsay Boeckman, Wendy Harrison, Andrea S. Winkler

**Affiliations:** 1 Department of Neurology, Center for Global Health, School of Medicine, Technical University of Munich, Munich, Germany; 2 Centre for Global Health, Institute of Health and Society, University of Oslo, Oslo, Norway; 3 Département de Pathologie et Microbiologie, Faculté de médecine vétérinaire, Université de Montréal, Saint-Hyacinthe, Canada; 4 Département de médecine sociale et préventive, École de santé publique de l’université de Montréal, Montréal, Canada; 5 Centre de Recherche en Santé Publique (CReSP) de l’université de Montréal et du CIUSS du Centre Sud de Montréal, Montréal, Canada; 6 Groupe de Recherche en Épidémiologie des Zoonoses et Santé Publique (GREZOSP), Université de Montréal, Saint-Hyacinthe, Canada; 7 Department of Anaesthesiology, University Hospital Würzburg, Würzburg, Germany; 8 Department of Neurology, University Hospital Erlangen, Erlangen, Germany; 9 Department of Radiology, University Hospital Tuebingen, Tuebingen, Germany; 10 National Epilepsy Association Malawi, International Bureau of Epilepsy, School of Health Social Sciences University of Edinburgh, Edinburgh, United Kingdom; 11 The University of Oklahoma Health Sciences Center, Oklahoma City, Oklahoma, United States of America; 12 Department of Infectious Disease Epidemiology, Imperial College London, London, United Kingdom; University of Catania, ITALY

## Abstract

**Background:**

Epilepsy and neurocysticercosis (NCC) prevalence estimates in sub-Saharan Africa are still scarce but show important variation due to the population studied and different screening and diagnosis strategies used. The aims of this study were to estimate the prevalence of epileptic seizures and epilepsy in the sampled population, and the proportion of NCC among people with epilepsy (PWE) in a large cross-sectional study in a rural district of southern Malawi.

**Methods:**

We conducted a community-based door-to-door screening study for epileptic seizures in Balaka, Malawi between October and December 2012. Past epileptic seizures were reported through a 15-item questionnaire answered by at least one person per household generating five major criteria. People who screened positive were further examined by a neurologist to establish diagnosis. Patients diagnosed with epilepsy were examined and offered *Taenia solium* cyst antigen and antibody serological tests, and a CT scan for the diagnosis of NCC.

**Results:**

In total, screening information on 69,595 individuals was obtained for lifetime occurrence of epileptic seizures. 3,100 (4.5%) participants screened positive, of whom 1,913 (62%) could be followed-up and underwent further assessment. Lifetime prevalence was 3.0% (95% Bayesian credible interval [CI] 2.8 to 3.1%) and 1.2% (95%BCI 0.9 to 1.6%) for epileptic seizures and epilepsy, respectively. NCC prevalence among PWE was estimated to be 4.4% (95%BCI 0.8 to 8.5%). A diagnosis of epilepsy was ultimately reached for 455 participants.

**Conclusion:**

The results of this large community-based study contribute to the evaluation and understanding of the burden of epilepsy in the population and of NCC among PWE in sub-Saharan Africa.

## Author summary

Epilepsy is more common in low-income and middle-income countries than in high-income countries. One of the reasons for this is that risk factors for acquired epilepsy such as *Taenia solium* cysticercosis are more common in these countries. In this study we conducted a screening for epileptic seizures among nearly 70,000 people of rural Balaka district of Malawi. We found that around 3 percent of people have ever experienced an epileptic seizure and that 1 percent of people suffer from epilepsy. The screening questionnaire that we used was quite accuracte, and the more criteria for screening positive were fulfilled, the more likely it was that the person has ever experienced an epileptic seizure. Among the patients with epilepsy, around 5% also had neurocysticercosis typical lesions in their brain.

## Introduction

Epilepsy is among the most common neurological disorders globally, with approximately 50 million people affected [1]. Prevalence varies considerably around the globe and 80% of affected individuals are assumed to live in low and middle-income countries (LMIC) [2,3]. This disparity is also due to a higher prevalence of risk factors for secondary epilepsy in LMIC. These include perinatal asphyxia and traffic-associated traumatic head injuries as well as parasitic infections. For example, neurocysticercosis (NCC) is suspected to be one of the leading causes of acquired epilepsy in many parts of the world, including sub-Saharan Africa [4–6].

NCC is an helminthic infection by the zoonotic tapeworm *Taenia solium*. Humans act as the definitive host of the parasite and infect pigs through eggs contained in their faeces; humans get infected again through consumption of undercooked pork containing parasitic larvae. Humans may also act as an accidental host when ingesting parasitic eggs. The larval stage (cysticercosis) in humans may have serious effects especially when established in the central nervous system. Subsequent immune response and inflammation in the brain may ultimately lead to neurological symptoms including epileptic seizures. In sub-Saharan Africa, up to 2.5 million people are estimated to suffer from epilepsy due to NCC [4]. In addition, up to 80% of NCC cases may be asymptomatic, as cysticerci can persist in the brain for many years without any inflammatory response [7,8].

It is known that anthelmintic drugs such as albendazole or praziquantel can cross the blood-brain barrier and kill viable *T*. *solium* larvae in the brain, making it a risk for provoking an exacerbation of NCC, potentially leading to severe neurological signs/symptoms including brain oedema and potential death [4]. That is why in NCC treatment the concomitant administration of steroids is recommended [9].

Anthelmintics are also used for treatment of other infections such as lymphatic filariasis, schistosomiasis and soil-transmitted helminths, ranking among the most prevalent neglected tropical diseases (NTD) worldwide [10]. In endemic areas, periodic mass drug administration (MDA) is frequently applied to control those infections. The World Health Organization (WHO) recommends that MDA be offered to large populations without individual diagnosis as a strategy to reduce the burden of several NTDs [11].

Yet, data from serological and epilepsy studies suggest that up to 8.2 million people may suffer from (usually undiagnosed) NCC in sub-Saharan Africa [4]. Consequently, there is a substantial risk of neurological side effects in the context of anthelmintic MDA, especially in co-endemic areas. As symptomatic NCC presents as epileptic seizures in most cases [12], people with active NCC (i.e. viable larvae in the brain) should be excluded from MDA to reduce the risk of major post-MDA neurological adverse events.

However, in sub-Saharan Africa, access to modern anti-seizure medicine is limited for many people and a lack of awareness and education concerning epileptic seizures and epilepsy combined with traditional beliefs and subsequent social stigma lead to underdiagnosis and a large treatment gap of the disease [13,14]. Hence, before implementing MDA, it is important to estimate what proportion of PWE are associated with NCC, which could indicate the extent to which a relatively large number of people are likely to have asymptomatic undiagnosed active NCC. In addition, it is important to identify people with active epilepsy since, if their epilepsy is due to NCC, they are more likely to still have active NCC lesions in their brain, putting them at higher risk of severe side effects following MDA treatment.

In epidemiological research on epilepsy in LMIC, screening questionnaires are typically used to identify possible PWE, which are then examined by a medical doctor to confirm (or not) the presence of epileptic seizures or epilepsy. Confirmed cases are used to obtain prevalence estimates in the area. Several tools have been introduced and have been validated in different populations, but not yet for the local languages (Chichewa, Chiyao) in Balaka district in Malawi which is challenging because of different cultural concepts regarding epileptic seizures [15]. So far, there exists no standard universal screening questionnaire for the identification of epileptic seizures and epilepsy in the general population, and both epilepsy and NCC estimates in sub-Saharan Africa are still scarce. Consequently, there is a need for additional large-scale studies.

The aim of this study was to examine prevalence proportions of epileptic seizures, epilepsy and NCC among PWE using a large project of MDA (with a praziquantel/albendazole) in a rural district in Malawi. Additionally, we aimed to assess the positive predictive value of a screening questionnaire for epileptic seizures.

## Methods

### Ethics statement

The study was approved by the following ethical committees: The Malawi National Health Science Research Committee (NHSRC Ref. No. 910); the Imperial College Research Ethics Committee (Ref. No. ICREC_11_3_6); and by the Ethics Committee of the Technical University of Munich (Ref. No. 3088/10). All individuals gave oral informed consent prior to inclusion in the study. For children under age, consent was obtained from the parent/guardian. For adjudication of diagnosis, clinical examination, serological testing and CT scanning, written informed consent was obtained. All patients diagnosed with epilepsy were followed-up by a neurologist several times for surveillance of medication, compliance, side effects and education about the diseases. Patients were referred to a non-governmental organisation (Sue Ryder) for clinical consultations after the end of the study. Furthermore, health officers of the villages were informed about all patients with epilepsy to assure continued clinical care.

For the reporting of the study findings, the STROBE guidelines were followed (Text B in S1 File).

### Study site

This analysis was conducted as part of a study designed to assess the safety and potential side effects of MDA. The study site was Balaka district in southern Malawi. This district was selected because *Schistosoma mansoni* seroprevalence was known to be >20%, no MDA programme had been conducted in the two previous years (albendazole or praziquantel), pigs were being kept in the region and were often roaming freely, and although unknown, *T*. *solium* was expected to be endemic due to the high prevalence of epilepsy. The study district consisted of two traditional authorities: Kalembo and Msamala. Within these, three health centres were used as location for neurological examination: Kalembo (serves a population of ~55,000), Mbera (serves a population of ~49,000), and Chiyendausiku (serves a population of ~13,000) for a total estimated population of the catchment area of ~117,000 people.

### Study procedures

The main study had two phases. In phase 1, a community-based door-to-door screening survey for epileptic seizures was conducted between October and December 2012 to find people with epileptic seizures so that they would be excluded from MDA for safety reasons. All people of a household were included irrespective of whether they were present or not. Present adults mostly answered for themselves. When an adult was absent, a parent, the head of household or the oldest person from the household present answered (in this order). For minors (<18 years), a parent or the head of household answered in most cases. In addition to the screening for epileptic seizures, demographics and data regarding pig keeping, pork consumption and knowledge about *T*. *solium* were recorded. Between January and April 2013, screen positives were further examined by a neurologist to establish a diagnosis. Once diagnosed with epilepsy, patients immediately underwent medical history taking including semiological characteristics, as well as physical and neurological examination. A blood sample was collected at the same time to assess the presence of cystic *T*. *solium* antigens and antibodies (see below for details). A CT scan was also offered to assess the presence of NCC or other lesions. CT scans were conducted between January and December 2013. All people who were not diagnosed with epilepsy (regardless of whether active or inactive) were eligible for MDA. This also included people who had epileptic seizures but were not diagnosed with epilepsy. MDA was given in December 2012 and January 2013. Phase 2 of the study was conducted post-MDA. All households were revisited between February and April 2013 using the same procedure as in phase 1; participants were asked whether they had received MDA and if they did, they were presented with a screening questionnaire for side effects, i.e. epileptic seizures, severe headache and hemiparesis. The study we present here, only covers data from Phase 1 of the main study: the screening for epileptic seizures and the establishing of the clinical diagnosis.

### Definition and measurement of epileptic seizures and epilepsy

Epileptic seizures and epilepsy were defined according to the International League against Epilepsy (ILAE) definition. A seizure was defined as a transient occurrence of signs/symptoms due to abnormal excessive or synchronous neuronal activity in the brain. Epileptic seizures were further categorised into provoked and unprovoked seizures, psychogenic seizures, seizures in childhood and single seizures. Epilepsy was defined as at least two unprovoked (or reflex) seizures occurring more than 24 hours apart. Active epilepsy was defined as the presence of epileptic seizures in the past five years; if the last epileptic seizure was longer ago, the patient was considered to have inactive epilepsy.

### Screening questionnaire for epileptic seizures

History of epileptic seizures was assessed using a 15-item questionnaire of which seven questions were used to determine five major criteria for epileptic seizures; with the remaining eight questions to gather more details. The eight remaining questions assessed the history of febrile seizures, additional focal/non-convulsive seizures, the frequency of seizures and the time since last seizure. A participant was defined as being screen positive if at least one of the five major criteria was fulfilled. The questionnaire was translated to Chichewa (local language) in writing and back-translated to English. The questionnaire was asked orally in Chichewa by a trained person who is proficient in the language. The screening questionnaire and the five major criteria can be found in Text A in S1 File [16].

### Clinical, serological and radiological assessment

**Fig 1:**
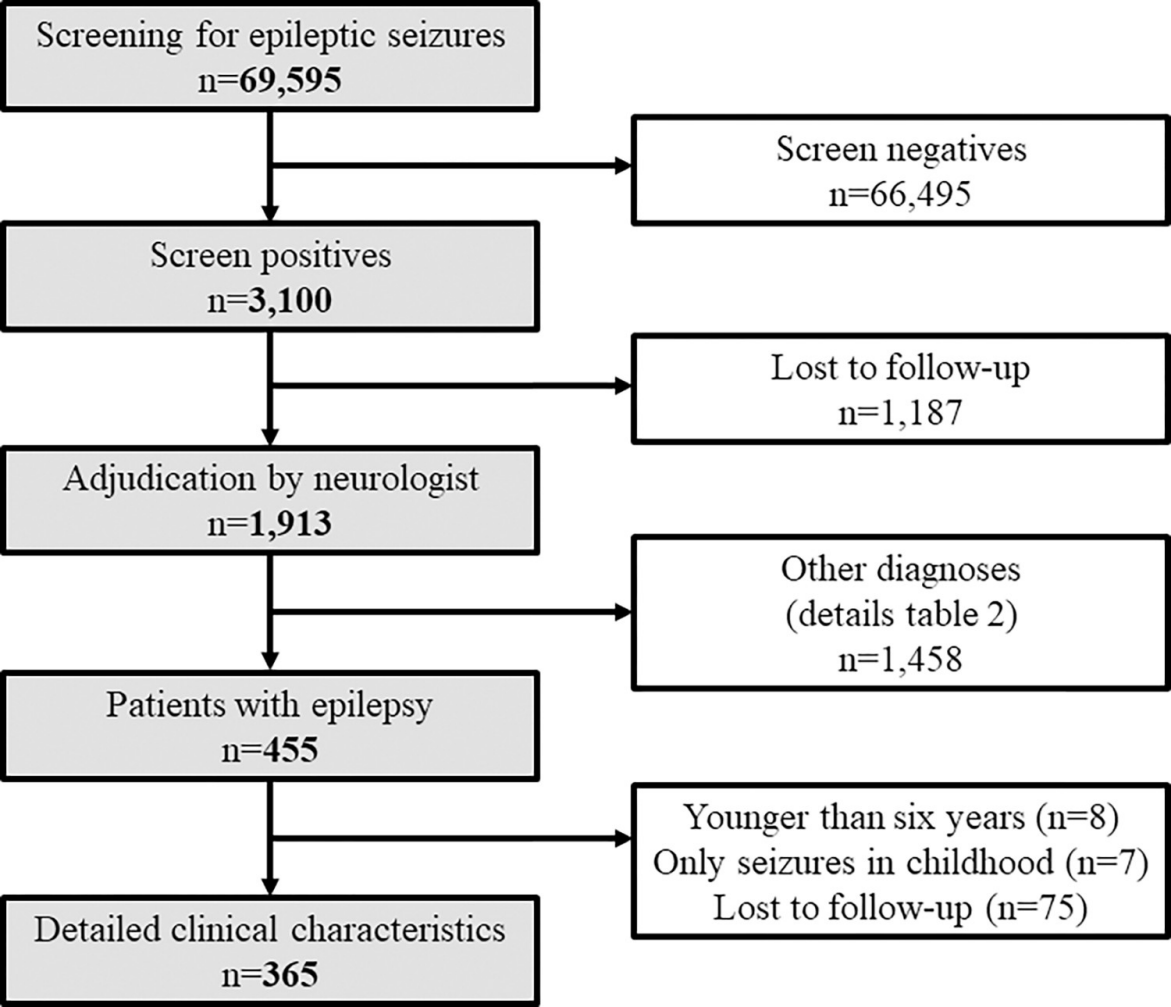
Flowchart of the study.

Screen positives were examined by the study neurologist (RLK). A lentil lectin purified glycoprotein enzyme-linked immunoelectrotransfer blot [LLGP-EITB] and rT24H-immunoblot were used to test for the presence of antibodies to the cystic form of *T*. *solium* [17,18] and for the presence of specific antigens with B158/B60-ELISA [19] among those with confirmed epilepsy. Serological tests were performed at the Division of Parasitic Diseases and Malaria, Center for Global Health, Centers for Disease Control and Prevention (CDC), Atlanta, Georgia, USA. If any test was positive, the patient was offered a CT scan (with and without contrast medium) for the diagnosis of NCC. For those who were negative in all three serological tests, all patients with seizure onset in the five years prior to the study were offered a CT scan and so were some randomly selected patients with earlier seizure onset (see flowchart Fig 1). CT scans were performed at Deayoung Hospital in Lilongwe; slice thickness of the CT scan was 6mm. Location of NCC lesions were categorised as parenchymal, extraparenchymal or mixed lesions; NCC lesions were furthermore categorised as active (i.e. vesicular, colloidal, granular-nodular stage), inactive or mixed stage. CT scans were evaluated by a neuroradiologist (VR). Diagnosis of NCC was based on the revised Del Brutto criteria [20].

### Statistical methods

The prevalence of screen positives was calculated by dividing the total number of people considered screened positive by the number of people for whom the screening questions were answered. The positive predictive value for the screening questionnaire was calculated by dividing the number of participants confirmed to have epilepsy by the total number of people screening positives. The unadjusted lifetime prevalence proportions of epileptic seizures and epilepsy were estimated by dividing the number of confirmed cases of epileptic seizures or epilepsy by the total number of people for whom the screening questionnaire was answered. The Pearson correlation coefficients between the village-level confirmed epileptic seizure prevalence with a) the distance to the closest main road, b) the proportion of households with free roaming pigs nearby and c) the Muslim population were calculated. We assessed these correlation coefficients to investigate factors that may influence prevalence of epileptic seizures (distance to closest main road as a proxy for household wealth and health service utilisation which may positively impact outcome of e.g. head trauma, or CNS infections; the other two as proxy for NCC as cause of epileptic seizures which may not have been detected in our assessment of NCC due to inaccuracy of serological tests or neuroimaging). The GPS coordinates for each village were determined by using the median latitude and longitude values of all individuals from the respective villages. The distance of the villages to the closest main road was analysed using the “geosphere” R package. The main roads were defined by open street map key “highway” and the values “motorway”, “trunk”, “primary” and “secondary”. The positive predictive value of the screening questionnaire for epileptic seizures and epilepsy was reported by question and by the number of criteria fulfilled for the total population and separately for people answering for themselves and for other people answering the questionnaire. We analysed difference between screen positives and negatives, and between screen positives who were followed-up and those who were not followed-up using Chi-square tests.

The unadjusted proportions of epileptic seizures, epilepsy and of active NCC among patients with confirmed epilepsy were first calculated. We then used two different analyses. In the first analysis, we accounted for loss-to-follow-up by using multiple imputation. In the second analysis, we conducted Bayesian latent class models to account not only for loss-to-follow-up, but also for verification and misclassification bias.

### Multiple imputation analysis

Three multiple imputation models were used for epileptic seizures, epilepsy and NCC for people considered losses-to-follow-up. The three models were run using the “mice” R package assuming missing-at-random processes. Two logistic regressions, one each for the epileptic seizures and epilepsy, were used to impute data among those screened positive but not seen by the neurologist using age, sex and the person who answered the questionnaire as the independent variables. A third multiple imputation model was used to impute data for NCC among people with epilepsy including age, sex and serological test result as independent variables of the logistic regression model.

### Bayesian latent class models

Bayesian latent class models for epileptic seizures in the absence of a gold standard were run to adjust for verification bias, losses-to-follow up at the verification stage and misclassification error of the screening questionnaire [21]. Verification bias occurs if participants offered a first test have different probabilities of being selected to obtain a second test; in our study, all participants had the screening test but only those screening positive were eligible to receive a neurological examination [22]. We applied the same methods as Sahlu et al. [23] in their analyses of the impact of imperfect screening tools on measuring epileptic seizures, with different informative priors. Two different models were run. In Model 1, we used a uniform distribution as a vague prior (Unif(0.5, 1)) for specificity since several symptoms can mimic seizures. A uniform vague prior ranging between 30% and 100% was used for sensitivity to account for under-reporting of symptoms since epilepsy is highly stigmatized in sub-Saharan Africa and some forms of focal epilepsy are difficult to identify [24]. For Model 2, informative priors of a sensitivity of 95% (standard deviation 1%, dbeta(450.3, 23.7)) and a specificity of 99.8% (standard deviation 0.2%, dbeta(497.004, 0.996)) were used based on a review of epilepsy screening questionnaires and expert opinion [15]. Different values for the validity of screening questionnaires have been reported but none fitted our questionnaire well because of either the questions or the way questions were asked (mostly to the persons themselves or their parents but seldomly to the head of household). Also, in the review by Keezer et al large heterogeneity of accuracy of screening questionnaires was reported [15]. Therefore, we chose a combination of screening validity values that seemed reasonably according to expert opinion (RK, DS, ASW). The posterior distribution for the adjusted prevalence of epileptic seizures was multiplied by the total sample population (n = 69,595) to yield a distribution of the total estimated number of people with epileptic seizures in the study population. The results of both Bayesian latent class models on epileptic seizures were multiplied with a beta-distributed prior of the observed proportion (and standard deviation) of patients with epilepsy among those with epileptic seizures (37.3%, dbeta(34.52055, 58.02785)) to yield the lifetime prevalence of epilepsy. For the estimation of the proportion of patients with NCC among PWE, verification bias was present too, because not everybody gave a blood sample and because only a few people with negative serology were selected for CT scan (they were selected at random) [22]. Furthermore, for the diagnosis of NCC, a combination of magnetic resonance imaging and CT are recommended. In this study, only CT scans were performed. To account for the inaccuracy of the CT scan, we ran two Bayesian latent class models using beta distributions as priors assuming a sensitivity of a) 50% (dbeta(49.5, 49.5)) and b) of 80% (dbeta(50.4, 12.6)) for the detection of NCC-typical lesions, combined in both models with a specificity of 99.8% (dbeta(8.98101, 0.00899)). The NCC prevalence among PWE was then multiplied with the observed proportion of active stage lesions (60%, dbeta(57, 38)) among people with NCC to yield the prevalence of active stage NCC among PWE.

All Bayesian latent class models were run with 10,000 iterations of 4-chain models with a 2,000 iteration burn-in per chain. When modelling epileptic seizures, we used a vague Unif(0, 0.3) for the prevalence of epileptic seizures. We used JAGS and the r package “rjags” [25] for all the Bayesian analyses and reported the posterior mean and 95% Bayesian credible intervals (BCI). Convergence was assessed using traceplots. All statistical analyses were performed using R version 4.1.1.

## Results

### Screening

**Fig 2:**
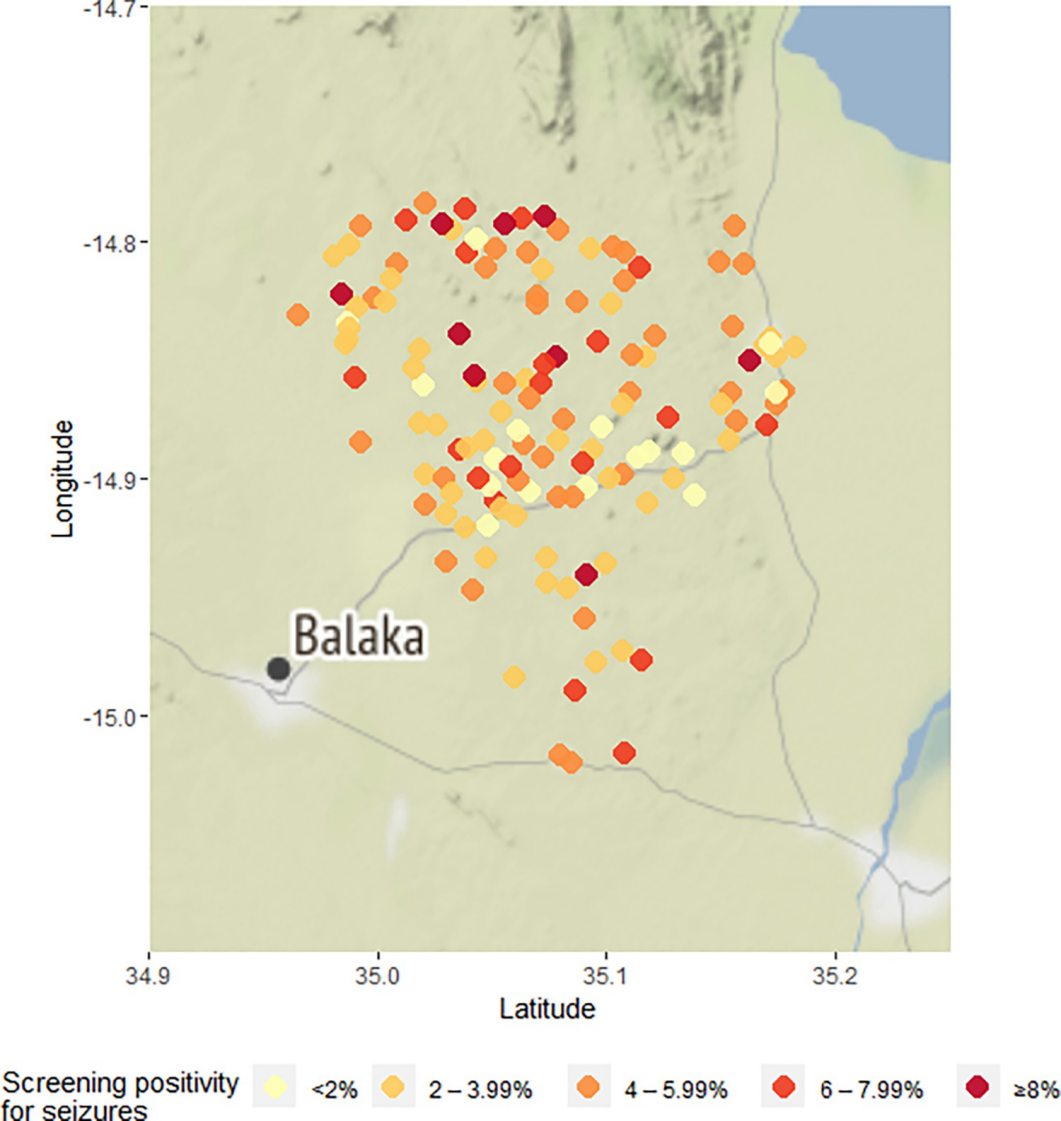
Villages by screening positivity for epilpetic seizures, Balaka district, Malawi.

Overall, the screening questionnaire was answered regarding 69,595 participants (Fig 1). Participants included 32,640 (47%) males, had a median age of 15 years (interquartile range 7 to 31 years) and lived in 16,062 households. More than half of all participants resided in Kalembo district (55%), 30% resided in Mbera and 16% in Chiyendausiku district. The mother answered for 46% of the participants, and these were largely children (89%), and 27% of participants answered for themselves. Pork consumption was reported for about a quarter of the participants, with most consumption occurring rarely. The sight of pigs roaming was reported for 21% of households, mostly in the village and rarely around their own home (Table 1). Overall, 3,100 (4.5%) of participants screened positive for epileptic seizures, with a similar prevalence across age groups, but with a peak between 6 and 17 years of age. The prevalence of screen positive was highest when the mother answered the questionnaire (5.3%) but lowest when it was the head of the household doing so (2.9%). Christians more commonly screened positives than Muslims and people of other faith (p = 0.03, Table 1). Eating pork and having had a tapeworm infection (either themselves or a family member) was associated with screening positive for epileptic seizures (p<0.001). Screening positivity varied considerably across villages; in nine of the 137 villages, screening positivity was ≥8%, and in 4 villages positivity was <1% (Fig 2).

**Table 1:** Baseline and demographic characteristics according to the result of the screening questionnaire used in a cross-sectional study conducted among a rural population of the Balaka district of Malawi, October to December 2012.

		Screening population(n = 69,595)		Screen positive	Screen positivity
		Frequency (%)	n	n	%
Total		69,595		3,100	4.5%
Who answered the questionnaire?	Self	18,728 (27)	69,560	720	3.8%
	Head of household	6,374 (9)		187	2.9%
	Mother	31,940 (46)		1,682	5.3%
	Father	2,606 (4)		107	4.1%
	Other	9,912 (14)		268	4.1%
District health centre	Chiyendausiku	10,515 (15)	69,157	560	5.3%
	Kalembo	37,964 (55)		1,614	4.3%
	Mbera	20,678 (30)		920	4.4%
Sex	Male	32,640 (47)	69,439	1,489	4.6%
	Female	36,799 (53)		1,611	4.4%
Age group (in years)	<11	26,394 (36)	67,524	1,386	5.3%
	11–17	11,506 (18)		609	5.3%
	18–35	15,970 (25)		630	3.9%
	>35	13,654 (20)		450	3.3%
Tribe	Yao	32,533 (47)	69,560	1,347	4.1%
	Lomwe	14,855 (21)		707	4.8%
	Ngoni	14,803 (21)		694	7.6%
	Sena	722 (1)		41	5.6%
	Mang’anja	584 (1)		46	7.9%
	Chewa	2,497 (4)		108	4.3%
	Other	3,566 (5)		157	4.4%
Religion	Christian	39,622 (57)	69,355	1,863	4.7%
	Muslim	29,671 (43)		1,234	4.2%
	Other or none	62 (0)		1	1.6%
Marital status	Married	21,546 (32)	66,770	660	3.1%
	Single	40,326 (60)		2,079	5.2%
	Widow	2,332 (3)		77	3.3%
	Divorced	2,566 (4)		128	5.0%
Pork consumption	No	50,802 (74)	69,116	2,119	4.2%
	Yes	18,314 (26)		978	5.3%
Frequency of pork consumption	Every day	21 (0)	18,239	0/978	
	Once a week	427 (2)		29/978	
	Once a month	2,964 (16)		169/978	
	Seldom	14,827 (81)		775/978	
	NA	75 (0)		5/978	
Pork consumption in the family	No	49,580 (72)	69,029	2,024	4.1%
	Yes	19,449 (28)		1,073	5.5%
Ever seen white spotted meat where buying meat	No	66,938 (97)	68,974	2,962	4.4%
	Yes	2,036 (3)		133	6.5%
Are there free roaming pigs around?	No	56,445 (82)	68,984	2,381	4.2%
	Yes	12,539 (18)		716	5.7%
Are there free roaming pigs around? By household	No	12,666 (79)	16,062		
	Yes	3,396 (21)			
Location of free roaming pigs	Own house	1,078 (9)	12,539	78/716	
	Neighbour	2,648 (21)		159/716	
	Village	8,758 (70)		475/716	
	NA	55 (0)		4/716	
Ever had tapeworm infection (respondent or family member)	No	65,052 (94)	68,966	2,625	4.0%
	Yes	3,914 (6)		472	12.1%

### Confirmation of diagnosis by a neurologist and lifetime prevalence of epileptic seizures and epilepsy

Of the 3,100 people who screened positive, less than two-thirds (1,913 or 62%) were examined (Fig 1). The remaining 1,187 (38%) were lost-to-follow-up. People who were not examined differed from people who were examined (people who were not examined were younger, more often residing in Kalembo district, fulfilled on average fewer criteria, particularly criteria 3 to 5, Tables A and B in S1 File). Of those examined, 1,217 (63.6% (95%CI 61.5 to 65.8%)) were confirmed to have a history of epileptic seizures among whom 455 were diagnosed as having epilepsy (23.8% (95%CI 21.9 to 25.7%). Most (402, 88%) were considered as active epilepsy cases (Table 2). The two approaches yielded fairly different estimates of lifetime prevalence of epileptic seizures with an estimated 2.9% (95%CI 2.8 to 3.0%) when the multiple imputation models approach (that only took into account loss-to-follow-up) was used and from 3.0% (95% Bayesian credible interval [BCI] 2.8 to 3.1%) to 5.3% (95%BCI 2.9 to 9.1%) using the Bayesian latent class models with informative or vague priors, respectively. Likewise, the lifetime prevalence of epilepsy varied considerably between the models and was 0.8% (95%CI 0.8 to 0.9%) in the multiple imputation model and from 1.2% (95% BCI 0.9 to 1.6%) to 3.4% (95%BCI 1.4 to 6.4%) in the two Bayesian latent class models (Table 2).

**Table 2:** Diagnoses of people screening positive in a questionnaire on epileptic seizures in a rural population in Balaka district, Malawi, October to December 2012.

	Examined (n = 1,913) n (%)
Active epilepsy	402 (21)
Inactive epilepsy	53 (3)
Epileptic seizures, but not epilepsy	750 (39)
*provoked seizures*	*29/750*
*seizures in childhood*	*715/750*
*single seizure*	*6/750*
Psychogenic seizures	12 (1)
Syncope	57 (3)
Loss of consciousness only	51 (3)
Hemiparesis	6 (0)
Other neurological disorders	73 (4)
Medical diagnoses	64 (3)
*general body weakness*	*6/64*
*arterial hypertension*	*33/64*
*anaemia*	*3/64*
*malnutrition*	*5/64*
*other*	*17/64*
Malaria	144 (8)
Psychiatric diagnoses	12 (1)
Orthopaedic diagnoses	13 (1)
Healthy	275 (14)
Correct screening for seizures	1,205
PPV in % (95%CI)	63.0% (60.9–65.2%)
Lifetime prevalence of epileptic seizures (raw; 95%CI)	2.8% (2.7–3.0%)
Lifetime prevalence of epileptic seizures (imputed; 95%CI)	2.9% (2.8–3.0%)
Lifetime prevalence of epileptic seizures (post-stratified–vague priors; 95%BCI)^⸸^	5.3% (2.9–9.1%)
Lifetime prevalence of epileptic seizures (post-stratified–informative priors; 95%BCI)^⸸^	3.0% (2.8–3.1%)
Estimated number of people with epileptic seizures in study population (post-stratified–informative priors; 95%BCI)^⸸^	2,058 (1,953–2,171)
Correct screening for epilepsy	455
PPV (95%CI)	23.8% (21.9–25.7%)
Lifetime prevalence of epilepsy (raw; 95%CI)	1.1% (1.0–1.1%)
Lifetime prevalence of epilepsy (imputed; 95%CI)	0.8% (0.8–0.9%)
Lifetime prevalence of epilepsy (post-stratified–vague priors; 95%BCI)^⸸^	3.4% (1.4–6.4%)
Lifetime prevalence of epilepsy (post-stratified–informative priors; 95%BCI)^⸸^	1.2% (0.9–1.6%)
Estimated number of people with epilepsy in study population(post-stratified–informative priors; 95%BCI)^⸸^	860 (642–1,099)

PPV positive predictive value; CI: Confidence interval; BCI: Bayesian credible interval

⸸ vague priors: sensitivity: unif(0.3,1), specificity: unif(0.5,1) informative priors: sensitivity (assumed screening accuracy for epileptic seizures: sensitivity = 95%; specificity = 99.8%; Proportion of epilepsy among patients with epileptic seizures (37.3%)

The number of criteria fulfilled in the screening question had an important impact on the PPV of the tool. The proportion of screened positive confirmed by the neurologist as having epileptic seizures increased from 44.6% with one criterion to 93.3% with 5 criteria met; the trends was even more marked for epilepsy with PPV increasing from from 5.4% with 1 criterion to 90% with all 5 criteria met. However, although the PPV of the tool increased with the number of criteria met, its sensitivity decreased considerably. If all five criteria had to be met, only 27 out of 455 people with epilepsy would have screened positive. When participants screened positive for only one criterion, the proportion confirmed as having epileptic seizure(s) depended on the criterion fulfilled. The proportion of screened positive confirmed with epileptic seizure(s) was highest for criteria 1 to 3 (Table 3). The proportion of screened positive confirmed with epileptic seizures was higher if another person answered the questionnaire. For epilepsy there was no association whether a person answered the questionnaire for himself/herself or not (Table B in S1 File).

**Table 3:** Epileptic seizure screening criteria and confirmation of epileptic seizure/epilepsy cases in a rural population in Balaka district, Malawi.

		Total	Epileptic seizures	Epilepsy
Overall	Overall	1913	1,205 (63.0)	455 (23.8)
Criterion⸸	1	1386	973 (70.2)	393 (28.4)
	2	1065	817 (76.7)	397 (37.3)
	3	468	321 (68.6)	93 (19.9)
	4	459	310 (67.5)	238 (51.9)
	5	408	314 (77.0)	243 (59.6)
Number of criteria fulfilled	1	836	373 (44.6)	45 (5.4)
	*Only criterion 1*	*429*	*222 (51*.*7)*	*22 (5*.*1)*
	*Only criterion 2*	*113*	*62 (54*.*9)*	*11 (9*.*7)*
	*Only criterion 3*	*138*	*69 (50*.*0)*	*4 (2*.*9)*
	*Only criterion 4*	*109*	*16 (14*.*7)*	*6 (5*.*5)*
	*Only criterion 5*	*47*	*4 (8*.*5)*	*2 (4*.*3)*
	2	508	341 (67.1)	86 (16.9)
	3	372	312 (83.9)	176 (47.3)
	4	167	151 (90.4)	121 (72.5)
	5	30	28 (93.3)	27 (90.0)^£^

⸸ More than one criterion was possible. For example, among the 1,386 people who screened positive for criterion 1, 957 screened positive for at least one other criterion; 429 only screened positive for criterion 1. The criteria can be found in S1 File.

£ Of the three participants without epilepsy, one had febrile seizures and two had cerebral malaria.

**Fig 3:**
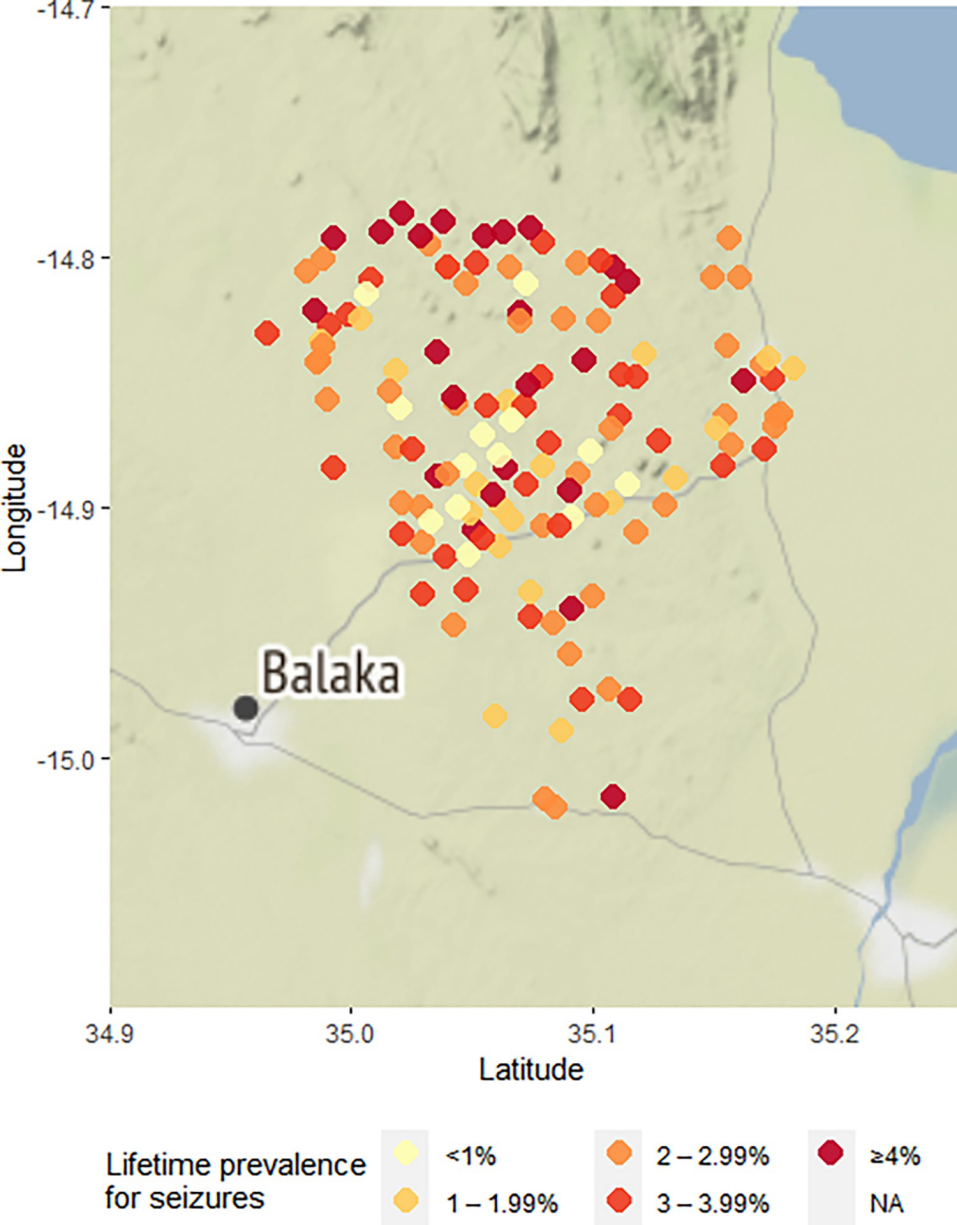
Villages by epileptic seizure prevalence, Balaka district, Malawi.

**Fig 4:**
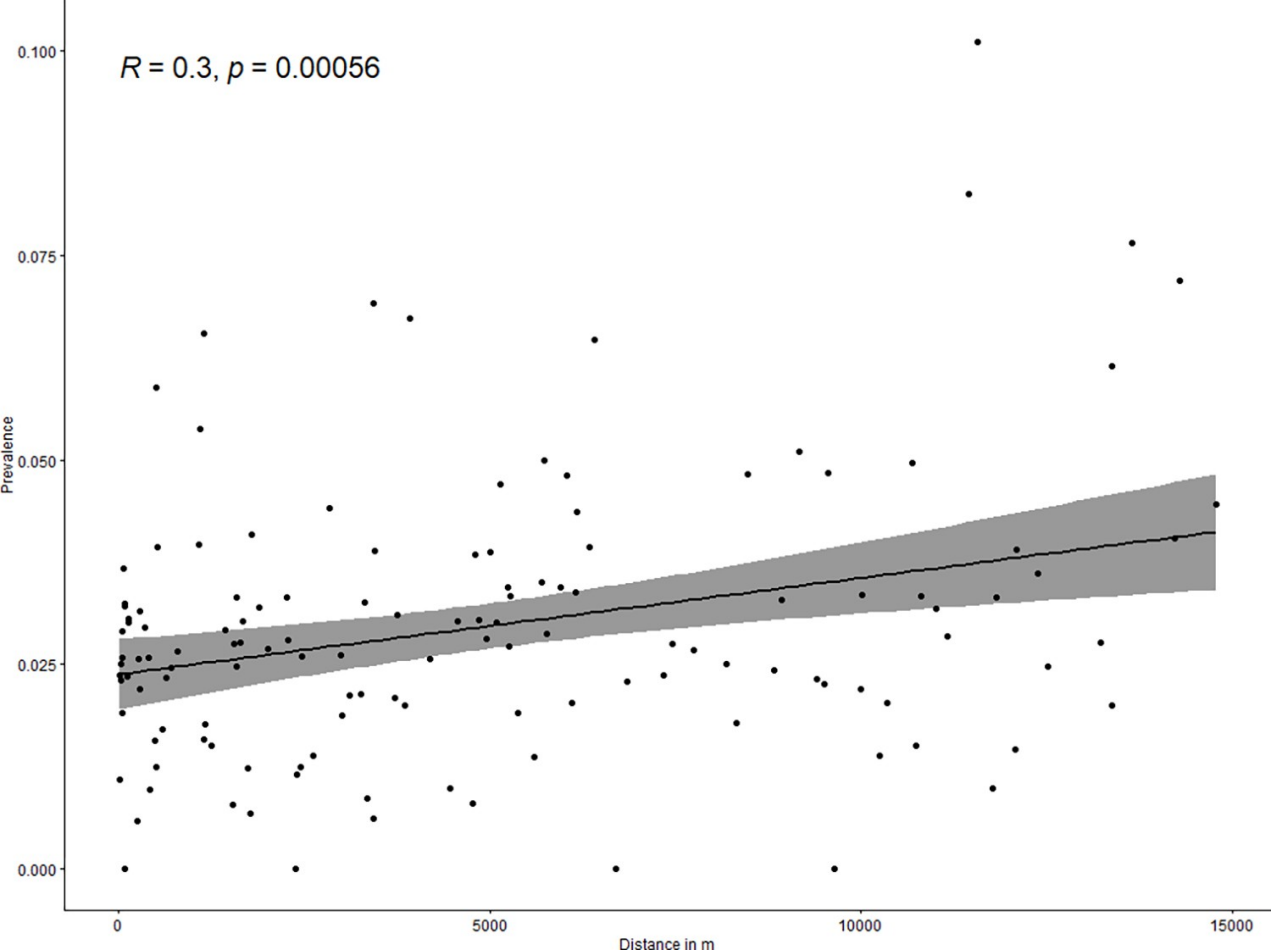
Scatterplot of distance to the closest main road and village seizure prevalence.

**Fig 5:**
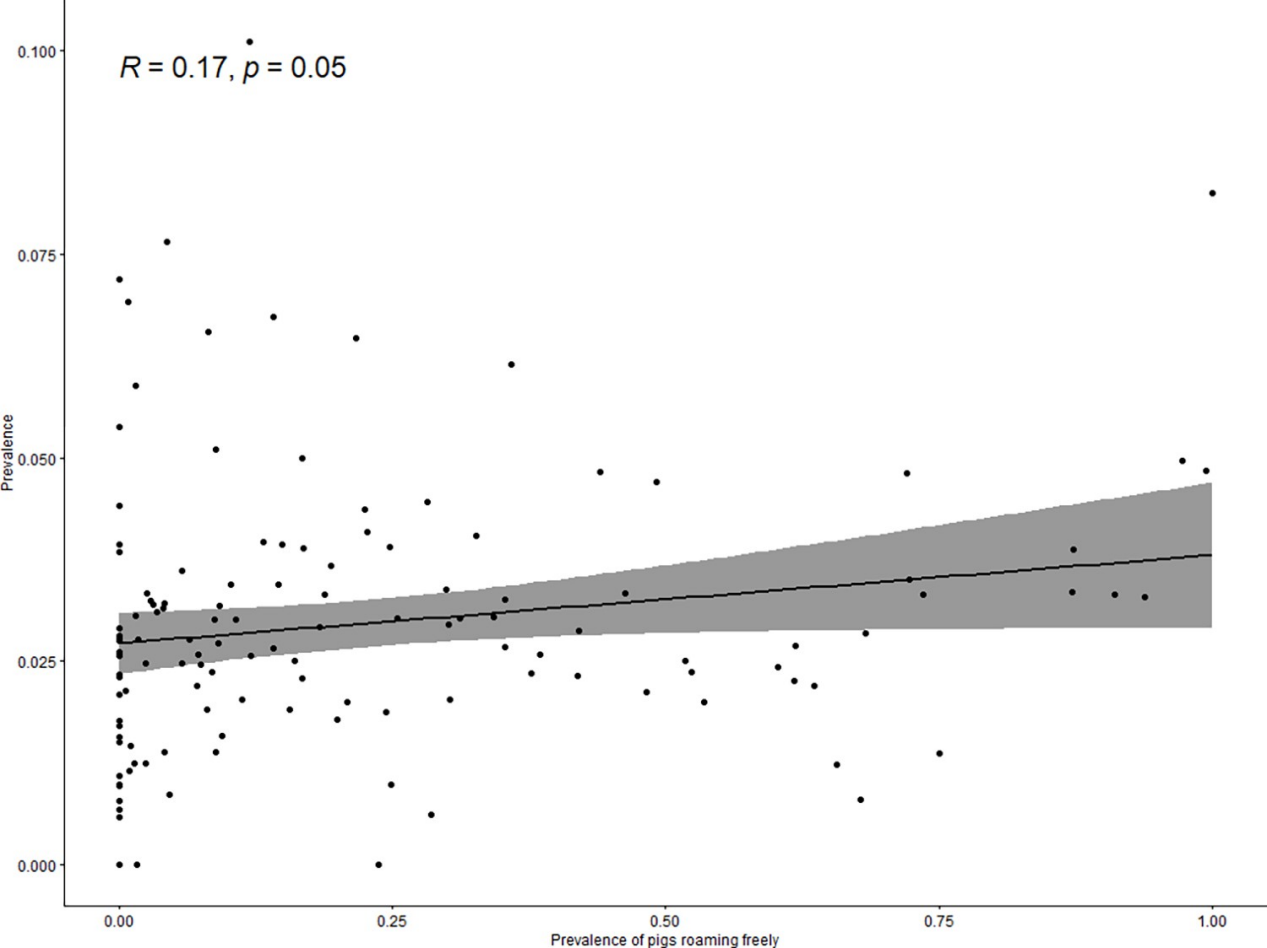
Correlation between proportion of free roaming pigs and village seizure prevalence.

**Fig 6:**
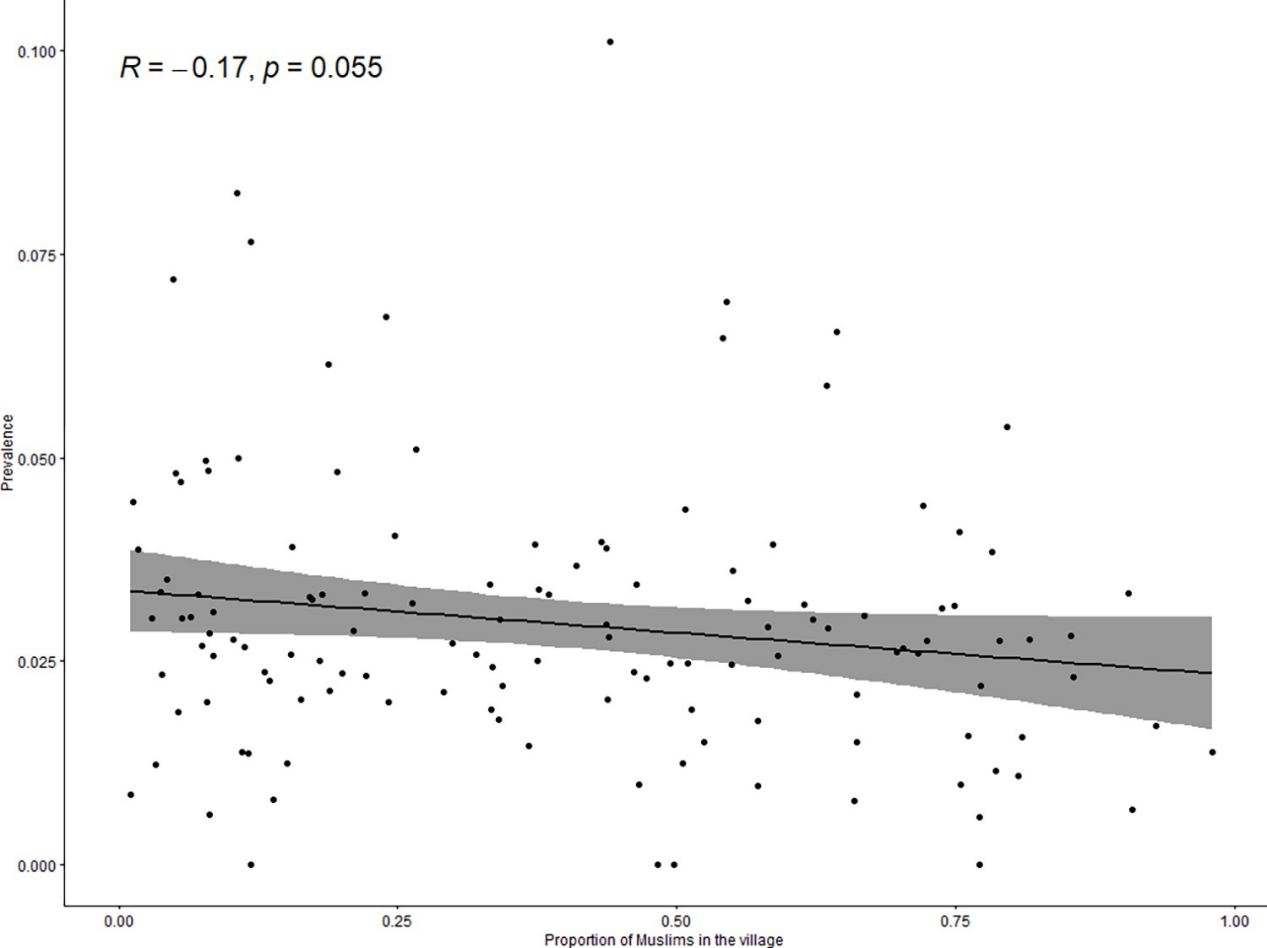
Correlation between proportion of Muslim population and village seizure prevalence.

The prevalence of epileptic seizures was ≥4% in 24 villages and <1% in 13 of the 137 villages (Fig 3). The prevalence of epileptic seizures at the village-level was positively correlated with distance to a main road (rho = 0.3, p<0.001; Fig 4), and with the proportion of households with free roaming pigs nearby (rho = 0.17, p = 0.05; Fig 5). The prevalence of epileptic seizures at the village-level was negatively correlated with the proportion of Muslims in a village (rho = -0.17, p = 0.055; Fig 6). All correlations were small.

Among the 1,913 screen positives examined by the neurologist, 708 (37%) had diagnoses other than epileptic seizures: e.g. 144 (7.5%) had a history of malaria, 12 (0.6%) had psychogenic seizures, 6 (0.3%) had a hemiparesis, 73 (3.8%) had other neurological disorders. A further 275 (14.4%) were healthy (Table 2).

### Clinical characteristics of people with epilepsy, serology and CT findings

**Fig 7:**
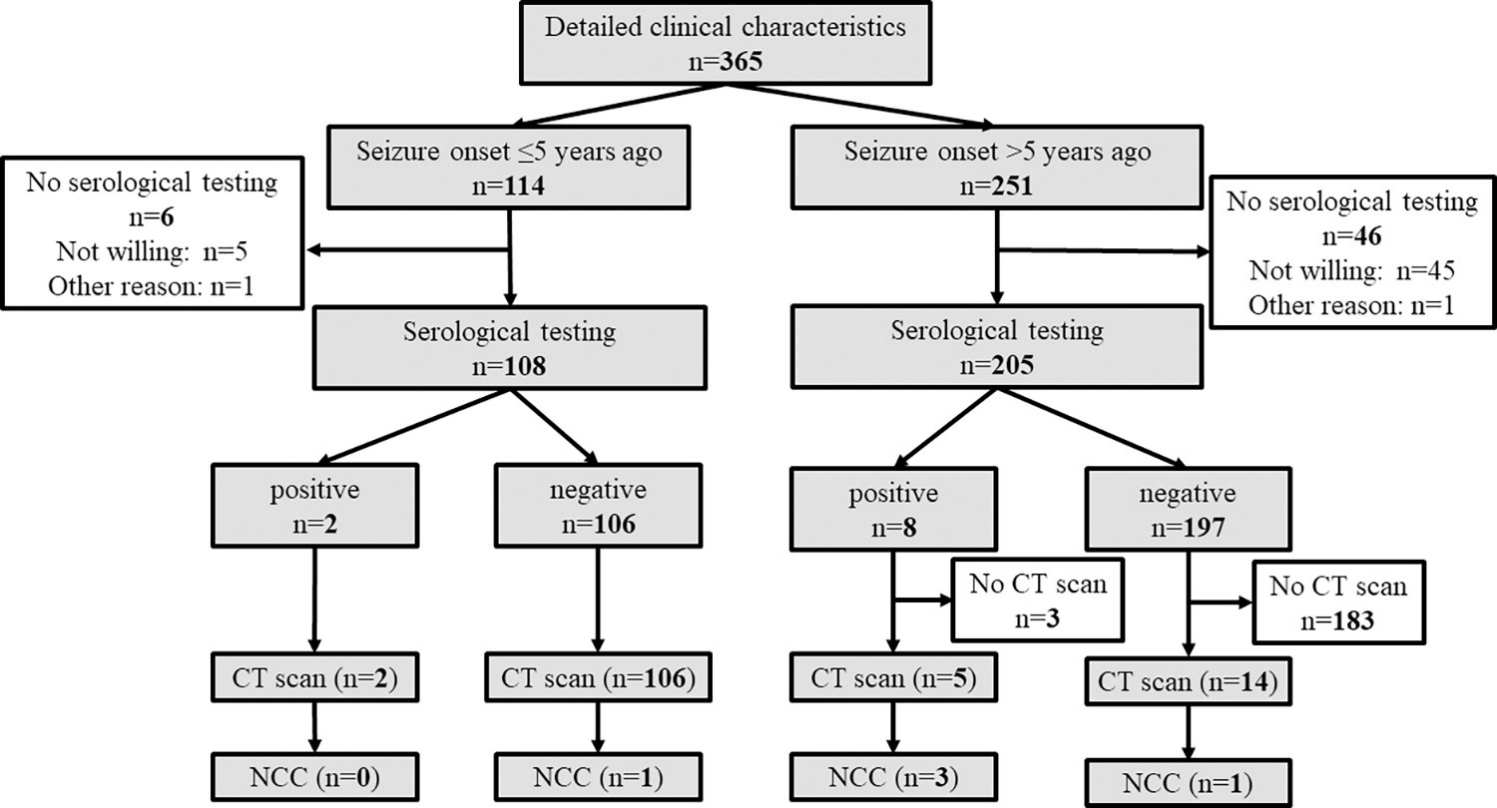
Flowchart of the people with epilepsy with detailed characteristics.

Of the 455 participants diagnosed with epilepsy, clinical characteristics were available for 365 (80%) patients. The median age of those 365 with full clinical information, was 21 years (interquartile range (IQR) 13 to 32 years) and 52% were female. The median age of seizure onset was 10 years old (IQR 6 to 20). Most PWE had generalised onset seizures (79%) and every other person reported at least one febrile seizure. The detailed clinical characteristics will be presented in a separate paper assessing the MDA data. The flowchart for selection for CT scanning is presented in Fig 7. Among 313 PWE who had serological testing performed, 10 were positive in at least one test (3.2%). Seven PWE with positive serology and another 120 randomly selected PWE with negative serology accepted to have a CT scan performed. Overall, five PWE had NCC of which three were in active or mixed stage, and two were in calcified stage. Four patients had a definite and one a probable NCC diagnosis according to Del Brutto. Three PWE with NCC only had parenchymal lesions, one only extraparenchymal lesions and one mixed parenchymal and extraparenchymal lesions. Four PWE had more than three lesions, and one person had a single lesion. Further, 11 PWE had post-ischaemic lesions on CT scan, nine had an atrophy, one had a post-haemorrhagic lesion, and one had an arachnoid cyst (Table C in S1 File). Overall NCC prevalence among PWE was 3.9% (95%CI 0.6–7.3%, 5/127), and 5.1% (95%CI 2.6–7.6%) after imputation of missing values (Table 4). Accounting for loss-to-follow-up, verification bias and the inaccuracy of the CT compared with a combination of CT and MRI, the NCC prevalence among PWE was 7.2% (95%BCI 1.4–14.3%) assuming a sensitivity of 50%, and 4.4% (95%BCI 0.8–8.5%) assuming a sensitivity of 80%, combined with a specificity of 98% in both cases. Two PWE with negative serology had NCC–one had five extraparenchymal active stage lesions and the other one had only one calcification (this patient was gp50 positive in the LLGP-EITB). Positive predictive values for NCC ranged between 40% and 42.5% for the different serological tests; negative predictive values ranged between 97.4% and 98.3% (Table 4).

**Table 4:** NCC diagnosis and serological findings among patients with epilepsy in rural Balaka district, Malawi.

		NCC	Comment
		5/127	NCC prevalence (raw):4.0% (95%CI 0.6–7.3%)
			NCC prevalence (data imputed):5.1% (95%CI 2.6–7.4%)
			NCC prevalence (post-stratified–informative priors)^⸸^:7.2% (95%BCI 1.4–14.4%)
			NCC prevalence (post-stratified–informative priors)^£^:4.4% (95%BCI 0.8–8.5%)
			Active NCC prevalence (post-stratified–informative priors)^⸸^:4.1% (95%BCI 1.6–8.2%)
			Active NCC prevalence (post-stratified–informative priors)^£^:2.5% (95%BCI 0.9–5.1%)
Serology	any positive	3/7	PPV: 42.9% (6.2–79.5%)
	all negative	2/120	NPV: 98.3% (96.1–100%)
LLGP-EITB	positive	3/7	PPV: 42.9% (6.2–79.5%)
	negative	2/120	NPV: 98.3% (96.1–100%)
rT24H-EITB	positive	2/5	PPV: 40% (0–82.9%)
	negative	3/122	NPV: 97.6% (94.8–100%)
Antigen ELISA	positive	2/5	PPV: 40% (0–82.9%)
	negative	3/122	NPV: 97.6% (94.8–100%)

⸸ assumed accuracy of CT: sensitivity = 50%, standard deviation 5%; specificity = 98%, standard deviation = 1%; Proportion of active NCC among NCC: 60%, standard deviation 5%

£ assumed accuracy of CT: sensitivity = 80%, standard deviation 5%; specificity = 98%, standard deviation = 1%; Proportion of active NCC among NCC: 60%, standard deviation 5%

PPV: positive predictive value; NPV: negative predictive value; NA not available; CI: Confidence interval; BCI: Bayesian credible intervals

The map was created using the ggmap library in R (D. Kahle and H. Wickham. ggmap: Spatial Visualization with ggplot2. The R Journal, 5(1), 144–161. URL: http://journal.r-project.org/archive/2013-1/kahle-wickham.pdf). Map tiles are by Stamen Design. Data are by OpenStreetMap

The map was created using the ggmap library in R (D. Kahle and H. Wickham. ggmap: Spatial Visualization with ggplot2. The R Journal, 5(1), 144–161. URL: http://journal.r-project.org/archive/2013-1/kahle-wickham.pdf). Map tiles are by Stamen Design. Data are by OpenStreetMap

## Discussion

In this large-scale community-based door-to-door study including nearly 70,000 individuals in rural southern Malawi, we examined the prevalence of epileptic seizures, epilepsy and NCC among PWE. We furthermore assessed a screening questionnaire for the detection of epileptic seizures.

Lifetime prevalence of epileptic seizures was calculated to be 2.8% (2.9% accounting for loss to follow-up and between 3.0% and 5.3% further accounting for verification and misclassification bias) for epileptic seizures and 1.1% (0.8% accounting for loss-to-follow-up, and between 1.2% and 3.4% further accounting for verification and misclassification bias) for epilepsy, respectively. These results are comparable to other African studies reporting lifetime epileptic seizure prevalence estimates of about 3% [26,27]. These findings are also line with a recent meta-analysis for sub-Saharan Africa revealing a lifetime prevalence for epilepsy of 1.6% and a prevalence for active epilepsy of 0.9% [28]. Another review including 119 epidemiological studies in sub-Saharan Africa also reported a median epilepsy prevalence of about 9.4 per 1,000 people [24], 12.9 for door-to-door studies, but only 5.1 per 1,000 for studies from eastern Africa (with large heterogenity between studies I^2^ = 0.967). Among the largest studies included were two door-to-door surveys from Kilifi district in Kenya [29,30]. Both used a 3-stage procedure for the diagnosis of epilepsy–in stage one heads of households were interviewed about whether any resident had had convulsions; in stage two, lay field workers interviewed individuals who screened positive in stage one, and in stage three a clinician examined those who screened positive in stage two for the final diagnosis [29,30]. One study yielded a prevalence of active convulsive epilepsy of 4.5 per 1,000 people and the other one a prevalence of 7.8 per 1,000 people. These estimates were adjusted for attrition and sensitivity of the questionnaire. In the latter study, sensitivity of the stage one questionnaire was assumed to be 100%. In both studies, considerable heterogeneity of prevalence of epilepsy and associated risk factors was observerd within the district. One reason why the epilepsy prevalence in our study was higher may be because we used a two-stage instead of a three-stage design. This means, in our study, every individual was screened for epileptic seizures and not only those who were pre-selected according to the answers by the household head; and as we were able to show, the likelihood of screening positive depends on who answers the questionnaire. This was also reported in a study from rural Mexico where a screening questionnaire answered by household members themselves detected more people with epilepsy than that answered by the household head [31]. Also, the assumption of 100% sensitivity of stage one in the study by Edwards et al [30] means that the chance of false negatives was excluded a priori which resulted in underestimated prevalence proportions. The prevalence we report, ranges within the reported prevalence for epilepsy in LMICs, being 2–3 times higher compared with high-income countries [2,32].

Amongst others, one cause of higher epilepsy prevalence in LMIC are neurological infections, e.g. epileptic seizures occur in about 80% of patients with symptomatic NCC [12]. NCC infection is associated with consumption of pork and also contact to free-roaming pigs reflecting a possible explanation for the lower epileptic seizure prevalence proportions in Muslims compared to Christians in our study [24,33]. Nevertheless, clinical assessment of PWE in our study population of Balaka district, yielded a rather low prevalence of NCC (less than 6%). Other studies in endemic areas including sub-Saharan Africa reported significantly higher prevalence estimates of symptomatic NCC in PWE ranging up to 20–40%, varying with geographic regions and the method of diagnosis [4,13,34]. Reasons for this low prevalence may be that the Muslim population in the district was higher than anticipated and pork consumption was lower than anticipated. Furthermore, reportedly only piglets until 1 to 2 months of age roam freely in the district. Thereafter, they are being tethered. Other risk factors such as open defaecation or eating of undercooked pork are reportedly not very common in the district. In our study, a combination of serological testing and CT scan was performed for diagnosis, according to the current standard criteria [20]. Serological testings showed negative predictive values of about 98% but only moderate values for positive prediction of NCC, a phenomenon commonly observed in *T*. *solium* endemic areas [33,35]. The data collection for this study took place 10 years ago, and it is undeniable that the epidemiological situation might have evolved during this time span. However, we do not think that NCC prevalence will have changed considerably as pig keeping has not increased and reportedly there have been no marked changes in the consumption of pork. In this study, only a few patients were found to have active stage NCC based on CT-scan conducted several weeks to months after the epilepsy assessment. The difficulty of diagnosing NCC in LMIC means that MDA programmes need to be implemented with the possibility of adverse events in mind. Indeed, the Pan American Health Organization recently published guidelines for preventive chemotherapy for the control of Taenia solium taeniasis in which active surveillance of adverse events is recommended for at least three days following MDA [36].

Overall, 4.5% of the participants screened positive for epileptic seizures. Our questionnaire consisting of 5 major criteria revealed a PPV of 63.6%, which is rather high compared to similar screening tools [15]. It is of note that the more criteria were fulfilled, the more likely diagnosis was confirmed, leading to a positive predictive value of up to >90% if ≥ 4 criteria were fulfilled. Albeit the questionnaire was designed to detect epileptic seizures, the above-mentioned association was also true for epilepsy.

Approximately one third of people with a confirmed history of epileptic seizures were ultimately diagnosed as “people with epilepsy”. This is a phenomenon frequently observed in field epidemiological studies and is a result of the underlying definitions, as single epileptic seizures may occur due to various conditions (e.g. fever) or even spontaneously without underlying epilepsy (see also www.ilae.org) [27,37]. In our study, this discrepancy was mainly due to a high number of PWE suffering from seizures in childhood only, without actual hints for underlying persistent epilepsy, and consequently not particularly relevant for further evaluation in our NCC-specific setting.

### Strengths and limitations

To our knowledge, this is one of the largest studies assessing a screening questionnaire for epileptic seizures in Africa [15,24]. The study had a sample size of nearly 70,000 people who were screened for epileptic seizures and 1,913 people were interviewed by a neurologist for adjudication of diagnosis.

Additionally, applying a combination of answers from the screening questionnaire to create five major criteria for diagnosis of epileptic seizures may have allowed for a more comprehensive and accurate screening than single item screening alone. By using our questionnaire we could e.g. filter for febrile seizures or syncopes

Furthermore, we were also able to assess NCC prevalence among PWE unbiased from whether a person was attending a mental health clinic or not. This is especially of note concerning possible spectrum effects of epidemiological studies caused by the method of patient selection [15,38]. Results from studies performed among clinic- or hospital-based populations are commonly at risk of being affected by this selection bias as they only include PWE being ill enough to seek medical help. In our study, we addressed this issue by applying a community-based door-to-door design. Another strength of this study is that we took into account loss-to-follow-up, verification and misclassification bias for our prevalence estimates of epileptic seizures and epilepsy. Furthermore, we had gps locations available for every village which allowed us to show the spatial differences in prevalence of epileptic seizures.

This study also had several limitations. First, only screen positives underwent further examination. The fact that no subset of screen negatives was evaluated prevented us from assessing the sensitivity and specificity of our screening questionnaire. This is due to the present study being nested within a larger project on neurological side effects due to latent NCC after MDA for the control of schistosomiasis in areas co-endemic for NCC and schistosomiasis. Had it been a separate project, we would have also included screen negatives to properly assess the questionnaire. However, we tried to overcome this limitation by running Bayesian latent class models which took into account verification and misclassification bias.

Nevertheless, as stigmatizing cultural beliefs are widely prevalent in sub-Sharan Africa one could also suppose that PWE not answering truthfully due to potential fear of stigma and discrimination may contibute to this phenomenon. Consequently, also under-estimation of seizure prevalence may occur [39,40]. Although screening tools usually take special beliefs and translation issues into account, these effects cannot be completely ruled out [41].

A further limitation was the relatively large number of drop-outs due to e.g. difficulties finding people again because no official address or phone number was available, people lived too far from the CT centre or because of scepticism about the CT scanning. This may have affected our results (only 127 patients had a complete diagnostic set for NCC) but we tried to overcome this by imputing data on patients not further evaluated. Also, the selection of people with seizure onset less than five years ago for CT scanning may have influenced our NCC prevalence estimates since it would have pre-selected patients with active stage lesions and may have excluded some patients with lesions which would have taken many years to calcify and result in epileptic seizures [42].

As another limitation, focal and especially non-motor seizures are not primarily captured by our screening criteria. Epilepsy screeening tools usually focus on detection of generalized convulsive seizures, as these can be assessed more easily and accurately [43]. A recent study conducted in urban Tanzania showed a higher prevalence of epilepsy by applying a questionnaire containing questions specifically designed to identify focal seizures. Unfortunately, screen positives were not further evaluated in that study to confirm wether the person really had focal seizures. Additionnally, questions screening for focal and non-motor seizures often face the problem of being too unspecific [27]. Because of that and due to our NCC-specific study setting we focused on detection of recent generalized motor seizures (see major criteria) as generalized seizures and focal seizures with secondary generalization represent the most common seizure types experienced in people with NCC [44].

In the context of focusing on recent events, our screening questionnaire also included one question asking for “attacks in the past 4 weeks” (criterion 4). Unfortunately, this question did not specify the type of attack, potentially leading to inclusion of individuals with loss of consciousness due to any cause and other neurological deficits. However, in our dataset, there were not many participants who only screened positive for this criterion. Nonetheless, better specification of this criterion might have led to an even better positive predictive value of our screening tool.

## Conclusion

The current study on community-based prevalence of epileptic seizures/epilepsy is one of the largest ever conducted in sub-Saharan Africa. In this study, we used a specifically developed screening questionnaire for epileptic seizures and report lifetime prevalence for epileptic seizures and epilepsy in a rural area in Malawi. Additionnally, we assessed NCC prevalence among PWE in a community based setting. The results of our study contribute to the evaluation and understanding of the burden of epilepsy (liftetime prevalence of epilepsy around 1% and hence twice as frequent as in high-income countries) and the significance of NCC in PWE in Balaka district, Malawi. Our questionnaire represents an easy-to-use and promising screening tool not only for epidemiological research but also for identifying people with epileptic seizures/epilepsy and possible NCC in endemic areas before MDA.

## Supporting information

S1 File Text A. Criteria for screening for epileptic seizures. Table A. Comparison of screen positives who were examined and who were not. Table B. Seizure screening criteria by examined or not. Table C. Screening questionnaire by who answered within the household. Table D. People with epilepsy. Fig A. Trace plot of posterior prevalence (p) of Bayesian latent class models for epileptic seizures with informative priors. Text B. STROBE Statement—Checklist of items that should be included in reports of cross-sectional studies.
(DOCX)
