# Supplementary material for: Community-level prevalence of epilepsy and of neurocysticercosis among people with epilepsy in the Balaka district of Malawi: A cross-sectional study
Source: PLoS Negl Trop Dis. 2022 Sep 15;16(9):e0010675. doi: 10.1371/journal.pntd.0010675 (PMC9477368; doi:10.1371/journal.pntd.0010675)
Supplement: S1 File — Text A. Criteria for screening for epileptic seizures. Table A. Comparison of screen positives who were examined and who were not. Table B. Seizure screening criteria by examined or not. Table C. Screening questionnaire by who answered within the household. Table D. People with epilepsy. Fig A. Trace plot of posterior prevalence (p) of Bayesian latent class models for epileptic seizures with informative priors. Text B. STROBE Statement—Checklist of items that should be included in reports of cross-sectional studies. (DOCX) [file pntd.0010675.s001.docx]

- Supporting information -

**Community-level prevalence of epilepsy and of NCC among people with epilepsy in the Balaka district of Malawi: a cross-sectional study**

Luise Keller MD^1^*, Dominik Stelzle MD^1^*, Veronika Schmidt PhD^1,2^, Hélène Carabin DVM PhD^8,9,10,11^, Ann-Kristin Reinhold MD^1,3^, Claudius Keller^1^, Tamara M Welte MD^4^, Vivien Richter MD^5^, Action Amos^6^, Lindsay Boeckman^7^, Wendy Harrison PhD^12^, Andrea S Winkler PhD^1,2^

* Contributed equally

**Short title:** Epilepsy screening in Malawi

^1^ Department of Neurology, Center for Global Health, School of Medicine, Technical University of Munich, Germany

^2^ Centre for Global Health, Institute of Health and Society, University of Oslo, Norway

^3^ Department of Anaesthesiology, University Hospital Würzburg, Germany

^4^ Department of Neurology, University Hospital Erlangen, Germany

^5^ Department of Radiology, University Hospital Tuebingen, Tuebingen, Germany

^6^ National Epilepsy Association Malawi, International Bureau of Epilepsy, School of Health Social Sciences University of Edinburgh

^7^ The University of Oklahoma Health Sciences Center, Oklahoma, United States of America

^8^ Département de Pathologie et Microbiologie, Faculté de médecine vétérinaire, Université de Montréal, Saint-Hyacinthe, QC, J2S 2M2, Canada.

^9^ Département de médecine sociale et préventive, École de santé publique de l'université de Montréal, Montréal, QC, H3N 1X9, Canada.

^10^ Centre de Recherche en Santé Publique (CReSP) de l'université de Montréal et du CIUSS du Centre Sud de Montréal, Montréal, QC, H3N 1X9, Canada.

^11^ Groupe de Recherche en Épidémiologie des Zoonoses et Santé Publique (GREZOSP), Université de Montréal, Saint-Hyacinthe, QC, J2S 2M2, Canada.

^12^ Department of Infectious Disease Epidemiology, Imperial College London, United Kingdom

**Corresponding author:**

Prof. Andrea S Winkler MD, PhD

Department of Neurology

Klinikum rechts der Isar

Technical University Munich

Ismaninger Strasse 22

81675 Munich, Germany

tel.: +49/89/41406954 email: andrea.winkler@tum.de<mailto:>

Word count: 3939

Abstract word count: 299

Tables and Figures: 4 Tables, 3 Figures

**Supplementary Tables/Figures:**

Text A. Criteria for screening for epileptic seizures

| 1) The questions with the code 2.1 + 2.2 had to be answered with” yes” at the same time (fixed combination when both questions have been answered with “yes”)  2.1 Have you ever lost consciousness or fallen due to lost consciousness?  2.2 Have you ever been told that while you were unconscious your arms and legs shake or stretch out?  2) The question with the code 2.7 answered with “yes”  2.7 Have you ever been told that you have or had epilepsy or epileptic fits?  3) The questions with the code 2.9 + 2.10 answered in a fixed combination. 2.9 has to be answered “yes” AND 2.10 has to be answered “no”  2.9 Did you/your child have seizures between one month and 7 years of age?  2.10 Was there a fever with all of the attacks?  4) The question with the code 2.13 answered with “yes”  2.13 Have you had an attack in the last 4 weeks?  5) The question with the code 2.17 answered with “yes”  2.17 Do you take antiepileptic medication? |
| --- |

Table A. Comparison of screen positives who were examined and who were not

|  | | **Total screen positives** | **Screen positive and examined** | **Screen positive but not examined** | **p-value** |
| --- | --- | --- | --- | --- | --- |
| Total | | 3,100 | 1,913 | 1,187 |  |
| Who answered the questionnaire? | Self | 720 | 577 (30) | 143 (12) | <0.001 |
|  | Head of household | 187 | 132 (7) | 55 (5) |  |
|  | Mother | 1,682 | 925 (48) | 757 (64) |  |
|  | Father | 107 | 50 (3) | 57 (5) |  |
|  | Other | 268 | 147 (8) | 121 (10) |  |
| District health centre | Chiyendausiku | 560 | 389 (20) | 171 (14) | <0.001 |
|  | Kalembo | 1,614 | 927 (48) | 687 (58) |  |
|  | Mbera | 920 | 591 (31) | 329 (28) |  |
| Sex | Male | 1,489 | 877 (46) | 612 (52) | 0.002 |
|  | Female | 1,611 | 1036 (54) | 575 (48) |  |
| Age group (in years) | <11 | 1,386 | 691 (36) | 697 (59) | <0.001 |
|  | 11–17 | 609 | 365 (19) | 244 (21) |  |
|  | 18–35 | 630 | 465 (24) | 166 (14) |  |
|  | >35 | 450 | 370 (19) | 72 (6) |  |
| Religion | Christian | 1,863 | 1169 (61) | 694 (58) | 0.15 |
|  | Muslim | 1,234 | 742 (39) | 492 (41) |  |
|  | Other or none | 1 | 0 (0) | 1 (0) |  |
| Pork consumption | No | 2,119 | 1301 (68) | 818 (69) | 0.63 |
|  | Yes | 978 | 610 (32) | 368 (31) |  |
| Frequency of pork consumption | Every day | 0/978 | 0/610 | 0/368 |  |
|  | Once a week | 29/978 | 18/610 | 11/368 |  |
|  | Once a month | 169/978 | 102/610 | 67/368 |  |
|  | Seldom | 775/978 | 486/610 | 289/368 |  |
|  | NA | 5/978 | 4/610 | 1/368 |  |
| Pork consumption in the family | No | 2,024 | 1247 (65) | 777 (65) | 0.913 |
|  | Yes | 1,073 | 664 (35) | 409 (34) |  |
| Ever seen white spotted meat where buying meat | No | 2,962 | 1805 (94) | 1157 (97) | <0.001 |
|  | Yes | 133 | 105 (5) | 28 (2) |  |
| Are there free roaming pigs around? | No | 2,381 | 1447 (76) | 934 (79) | 0.057 |
|  | Yes | 716 | 464 (24) | 252 (21) |  |
| Location of free roaming pigs | Own house | 78/716 | 41/464 | 37/252 |  |
|  | Neighbour | 159/716 | 106/464 | 53/252 |  |
|  | Village | 475/716 | 314/464 | 161/252 |  |
|  | NA | 4/716 | 3/464 | 1/252 |  |
| Ever had tapeworm infection (respondent or family member) | No | 2,625 | 1620 (85) | 1005 (85) | 1 |
|  | Yes | 472 | 291 (15) | 181 (15) |  |

The p-values are derived from Chi-square tests

Table B. Seizure screening criteria by examined or not

|  |  | **Total** | **Examined** | **Not examined** | **p-value** |
| --- | --- | --- | --- | --- | --- |
| Overall |  | 3,100 | 1,913 | 1,187 |  |
| Criterion⸸ | 1 | 2,389 (77.2) | 1,386 (72.6) | 1,003 (84.6) | <0.001 |
|  | 2 | 1,925 (62.2) | 1,065 (55.8) | 860 (72.5) | <0.001 |
|  | 3 | 505 (18.1) | 468 (28.6) | 37 (3.2) | <0.001 |
|  | 4 | 567 (22.7) | 459 (30.8) | 108 (10.7) | <0.001 |
|  | 5 | 589 (19.1) | 408 (21.4) | 181 (15.3) | <0.001 |
| Number of criteria fulfilled | 1 | 1,256 (40.5) | 836 (43.7) | 420 (35.4) | <0.001 |
|  | 2 | 1,077 (34.7) | 508 (26.6) | 569 (47.9) |  |
|  | 3 | 539 (17.4) | 372 (19.4) | 167 (14.1) |  |
|  | 4 | 192 (6.2) | 167 (8.7) | 25 (2.1) |  |
|  | 5 | 36 (1.2) | 30 (1.6) | 6 (0.5) |  |

⸸ More than one criterion was possible. The criteria can be found in the Supplementary Information.

Table C. Screening questionnaire by who answered within the household

|  |  | **Self-answered** | | | **Answered by someone else** | | |
| --- | --- | --- | --- | --- | --- | --- | --- |
|  |  | **Screen positive** | **Seizures** | **Epilepsy** | **Screen positive** | **Seizures** | **Epilepsy** |
| Overall | Overall | 577 | 279 (48.4) | 151 (26.2) | 1336 | 926 (69.3) | 304 (22.8) |
| Criterion⸸ | 1 | 370 | 212 (57.3) | 134 (36.2) | 1016 | 761 (74.9) | 259 (25.5) |
|  | 2 | 283 | 202 (71.4) | 128 (45.2) | 782 | 615 (78.6) | 269 (34.4) |
|  | 3 | 133 | 72 (54.1) | 21 (19.8) | 335 | 249 (74.3) | 72 (21.5) |
|  | 4 | 158 | 82 (51.9) | 75 (47.5) | 301 | 228 (75.7) | 163 (54.2) |
|  | 5 | 108 | 78 (72.2) | 73 (67.6) | 300 | 236 (78.7) | 170 (56.7) |
| Number of criteria fulfilled | 1 | 303 | 87 (28.7) | 23 (7.6) | 533 | 286 (53.7) | 22 (4.1) |
|  | *Only criterion 1* | *129* | *38 (29.5)* | *12 (9.3)* | *300* | *184 (61.3)* | *10 (3.3)* |
|  | *Only criterion 2* | *39* | *20 (51.3)* | *7 (17.9)* | *74* | *42 (56.8)* | *4 (5.4)* |
|  | *Only criterion 3* | *61* | *25 (41.0)* | *2 (3.3)* | *77* | *44 (57.1)* | *2 (2.6)* |
|  | *Only criterion 4* | *61* | *4 (6.6)* | *2 (3.3)* | *48* | *12 (25.0)* | *4 (8.3)* |
|  | *Only criterion 5* | *13* | *0 (0.0)* | *0 (0.0)* | *34* | *4 (11.8)* | *2 (5.9)* |
|  | 2 | 131 | 71 (54.2) | 26 (19.8) | 377 | 270 (71.6) | 60 (15.9) |
|  | 3 | 90 | 71 (78.9) | 56 (62.2) | 282 | 241 (85.5) | 120 (42.6) |
|  | 4 | 48 | 46 (95.8) | 42 (87.5) | 119 | 105 (88.2) | 79 (66.4) |
|  | 5 | 5 | 4 (80.0) | 4 (80.0) | 25 | 24 (96) | 23 (92) |

⸸ More than one criterion was possible. For example, among the 370 people who answered for themselves and who screened positive for criterion 1, 241 screened positive for at least one other criterion; 129 only screened positive for criterion 1. The criteria can be found in the Supplementary Information.

Table D. People with epilepsy

| Variables | Level | Overall (%) |
| --- | --- | --- |
| n |  | 365 |
| Age group | 6–15 years | 121 (33) |
|  | 16–30 years | 144 (39) |
|  | 31–45 years | 59 (16) |
|  | 46–60 years | 21 (6) |
|  | 61–80 years | 17 (5) |
|  | >80 years | 3 (1) |
| Sex | female | 189 (52) |
|  | male | 176 (48) |
| Tribe | Chewa | 15 (4) |
|  | Lomwe | 84 (23) |
|  | Ngoni | 90 (25) |
|  | Nyanja | 25 (7) |
|  | Yao | 141 (39) |
|  | other | 10 (3) |
| Religion | Christianity | 212 (58) |
|  | Islam | 141 (39) |
|  | other | 12 (3) |
| Transport medium | bicycle | 69 (19) |
|  | vehicle | 2 (1) |
|  | walking | 293 (80) |
| Time to health centre in hours | median (IQR) | 1.0 [0.5, 2.0] |
| Serology | positive | 10 (3) |
|  | negative | 303 (97) |
| CT scan | NCC | 5/127 (4) |
|  | *Active/mixed lesions* | *3/5* |
|  | *Calcified lesions* | *2/5* |
|  | *Single lesion* | *1/5* |
|  | *2 or 3 lesions* | *0/5* |
|  | *>3 lesions* | *4/5* |
|  | *Parenchymal* | *3/5* |
|  | *Extraparenchymal* | *1/5* |
|  | *Parenchymal and extraparenchymal* | *1/5* |
|  | post-ischaemic lesion | 11/127 (9) |
|  | post-haemorrhagic lesion | 1/127 (1) |
|  | atrophy | 9/127 (7) |
|  | arachnoid cyst | 1/127 (1) |

Fig A. Trace plot of posterior prevalence (p) of Bayesian latent class models for epileptic seizures with informative priors


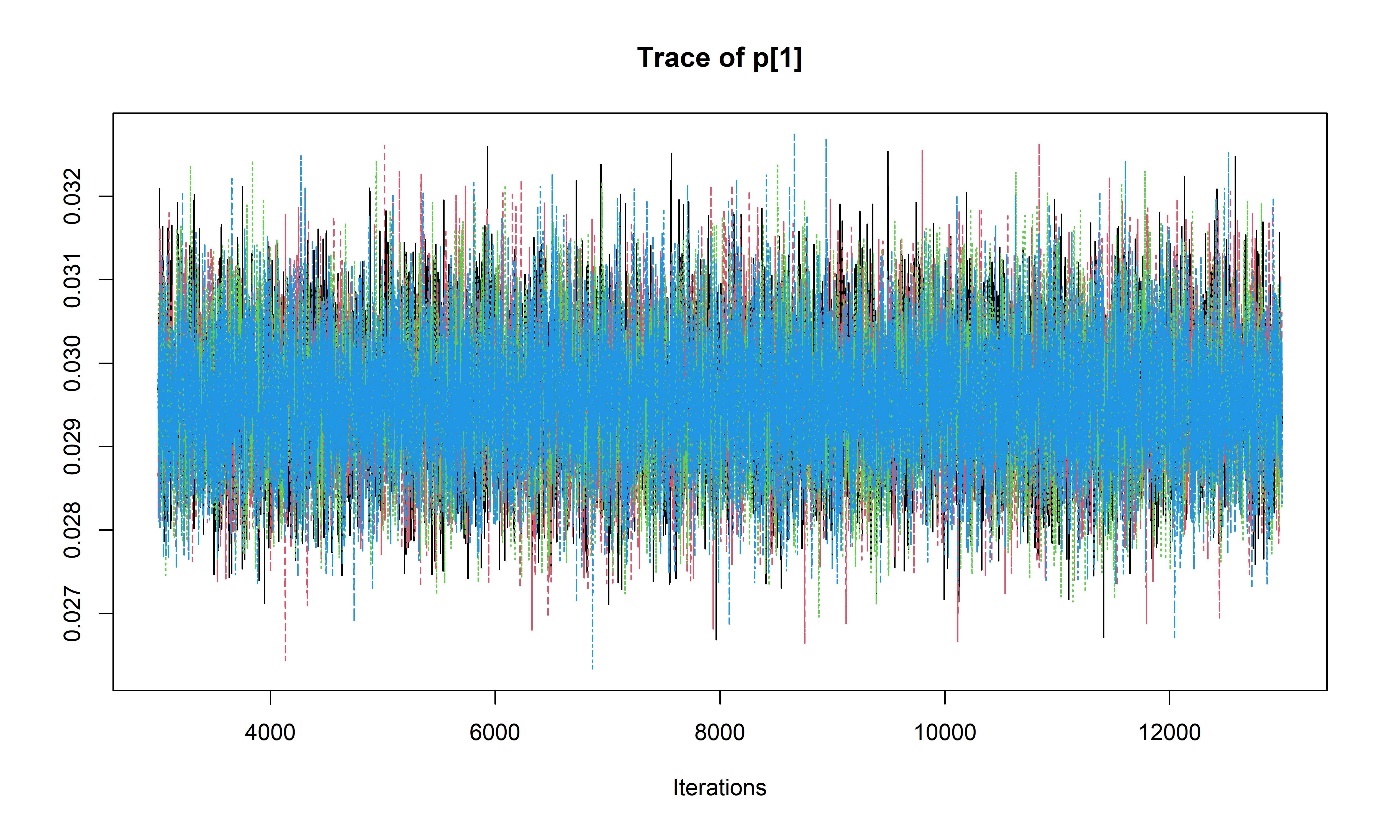


Text B. STROBE Statement—Checklist of items that should be included in reports of cross-sectional studies

|  | Item No | Recommendation | Location |
| --- | --- | --- | --- |
| **Title and abstract** | 1 | (*a*) Indicate the study’s design with a commonly used term in the title or the abstract | Title |
|  |  | (*b*) Provide in the abstract an informative and balanced summary of what was done and what was found | Abstract |
| Introduction | | |  |
| Background/rationale | 2 | Explain the scientific background and rationale for the investigation being reported | Beginning to last paragraph |
| Objectives | 3 | State specific objectives, including any prespecified hypotheses | Last paragraph |
| Methods | | |  |
| Study design | 4 | Present key elements of study design early in the paper | *“Study procedures”* |
| Setting | 5 | Describe the setting, locations, and relevant dates, including periods of recruitment, exposure, follow-up, and data collection | *“Study site”*, relevant dates under *“study procedures”* |
| Participants | 6 | (*a*) Give the eligibility criteria, and the sources and methods of selection of participants | *“Study procedures”; “Screening questionnaire for epileptic seizures*” and *“Clinical, serological and radiological assessment”* |
| Variables | 7 | Clearly define all outcomes, exposures, predictors, potential confounders, and effect modifiers. Give diagnostic criteria, if applicable | *“Definition and measurement of epilepsy and epileptic seizures”*; *“Supplementary Information. Criteria for screening for seizures”* |
| Data sources/ measurement | 8 | For each variable of interest, give sources of data and details of methods of assessment (measurement). Describe comparability of assessment methods if there is more than one group | See above under 7. |
| Bias | 9 | Describe any efforts to address potential sources of bias | *“Statistical analyses”* |
| Study size | 10 | Explain how the study size was arrived at | NA |
| Quantitative variables | 11 | Explain how quantitative variables were handled in the analyses. If applicable, describe which groupings were chosen and why | *“Statistical analyses”; “Definition and measurement of epilepsy and epileptic seizures”* |
| Statistical methods | 12 | (*a*) Describe all statistical methods, including those used to control for confounding | *“Statistical analyses”* |
|  |  | (*b*) Describe any methods used to examine subgroups and interactions | *“Statistical analyses”* |
|  |  | (*c*) Explain how missing data were addressed | *“Statistical analyses”* |
|  |  | (*d*) If applicable, describe analytical methods taking account of sampling strategy | NA |
|  |  | (*e*) Describe any sensitivity analyses | NA |
| Results | | |  |
| Participants | 13 | (a) Report numbers of individuals at each stage of study—eg numbers potentially eligible, examined for eligibility, confirmed eligible, included in the study, completing follow-up, and analysed | *“Screening”* and Figure 1. Flowchart, Figure 3. |
|  |  | (b) Give reasons for non-participation at each stage | Figure 1. Flowchart, Figure 3. |
|  |  | (c) Consider use of a flow diagram | Figure 1. Flowchart, Figure 3. |
| Descriptive data | 14 | (a) Give characteristics of study participants (eg demographic, clinical, social) and information on exposures and potential confounders | *“Screening”*, Table 1 |
|  |  | (b) Indicate number of participants with missing data for each variable of interest | Table 1 |
| Outcome data | 15 | Report numbers of outcome events or summary measures | Table 2, Table 3, Table 4 |
| Main results | 16 | (*a*) Give unadjusted estimates and, if applicable, confounder-adjusted estimates and their precision (eg, 95% confidence interval). Make clear which confounders were adjusted for and why they were included | Table 2, Table 4 |
|  |  | (*b*) Report category boundaries when continuous variables were categorized | NA |
|  |  | (*c*) If relevant, consider translating estimates of relative risk into absolute risk for a meaningful time period | NA |
| Other analyses | 17 | Report other analyses done—eg analyses of subgroups and interactions, and sensitivity analyses | *“Confirmation of diagnosis by a neurologist and lifetime prevalence of epileptic seizures/epilepsy”*, Supplementary Table 2, Supplementary Table 3 |
| Discussion | | |  |
| Key results | 18 | Summarise key results with reference to study objectives | First paragraph of the discussion |
| Limitations | 19 | Discuss limitations of the study, taking into account sources of potential bias or imprecision. Discuss both direction and magnitude of any potential bias | *“Strengths and limitations”* |
| Interpretation | 20 | Give a cautious overall interpretation of results considering objectives, limitations, multiplicity of analyses, results from similar studies, and other relevant evidence | Throughout the discussion section |
| Generalisability | 21 | Discuss the generalisability (external validity) of the study results | Conclusions |
| Other information | | |  |
| Funding | 22 | Give the source of funding and the role of the funders for the present study and, if applicable, for the original study on which the present article is based | Funding |
